# Development of a Radiomic-clinical Nomogram for Prediction of Survival in Patients with Nasal Extranodal Natural Killer/T-cell Lymphoma

**DOI:** 10.2174/0115734056319914250605053257

**Published:** 2025-06-19

**Authors:** Limin Chen, Zhao Wang, Xiaojie Fang, Mingjie Yu, Haimei Ye, Lujun Han, Ying Tian, Chengcheng Guo, Huang He

**Affiliations:** 1 Department of Medical Oncology, Sun Yat-sen University Cancer Center, State Key Laboratory of Oncology in South China, Collaborative Innovation Center for Cancer Medicine, Guangdong Provincial Clinical Research Center for Cancer, Guangzhou 510060, Guangdong, China; 2 Department of Radiology, Sun Yat-sen University Cancer Center, State Key Laboratory of Oncology in South China, Collaborative Innovation Center for Cancer Medicine, Guangdong Provincial Clinical Research Center for Cancer, Guangzhou 510060, Guangdong, China; 3 Department of Neurosurgery, Sun Yat-sen University Cancer Center, State Key Laboratory of Oncology in South China, Collaborative Innovation Center for Cancer Medicine, Guangdong Provincial Clinical Research Center for Cancer, Guangzhou 510060, Guangdong, China

**Keywords:** ENKTL, Radiomics, Magnetic resonance imaging, Prognosis, Lactate dehydrogenase, Intraclass Correlation Coefficient

## Abstract

**Introduction:**

An accurate and reliable prognostic model for Nasal Extranodal Natural Killer/T-cell Lymphoma (ENKTL) is critical for survival outcomes and personalized therapy. Currently, there is no Magnetic Resonance Imaging (MRI)- based radiomics analysis in the prognosis model for nasal ENKTL patients.

**Objective:**

We aim to explore the value of MRI-based radiomics signature in the prognosis of patients with nasal ENKTL.

**Methods:**

A total of 159 nasal ENKTL patients were enrolled and divided into a training cohort (n=81) and a validation cohort (n=78) randomly. Radiomics features from pretreatment MRI examination were extracted, respectively. Then two-sample t-test and Least Absolute Shrinkage and Selection Operator (LASSO) regression were used to select the radiomics signatures and establish the Rad-score. Univariate and multivariate Cox proportional hazards regression models were used to investigate the prognostic value of baseline clinical features and establish clinical models. A radiomics nomogram based on the Rad-score and clinical features was constructed to predict Overall Survival (OS). The predictive efficacy of the three models was evaluated in two cohorts.

**Results:**

A total of 1,345 features were extracted from T2-weighted (T2-w) and Contrast-enhanced T1-weighted (CET1-w) images, respectively, and 1,037 features with Intraclass Correlation Coefficient (ICC) >0.7 were selected. Ultimately, 20 features were chosen to construct the Rad-score, which showed a significant association with OS. The C-indexes of the Rad-score were 0.733 (95% confidence interval (CI): 0.645 to 0.816) and 0.824 (95% CI: 0.766-0.882), respectively, in training and validation cohorts. Through the univariate and multivariate analyses, three independent risk factors for OS were identified: Rad-score (HR: 10.962, 95% CI: 3.417-35.167, *P* <0.001), lactate dehydrogenase (LDH) level (HR: 3.009, 95% CI: 1.128-8.510, *P* = 0.028) and distant lymph-node involvement (HR: 2.966, 95% CI: 1.015-8.664, *P* = 0.047). Patients with distal lymph node involvement and LDH level before treatment were included in the clinical model, which achieved a C-index of 0.707 (95% CI: 0.600–0.814) in the training cohort and 0.635 (95% CI: 0.527–0.743) in the validation cohort.

We integrated the Rad-score and clinical variables to establish a radiomics nomogram, which exhibited a satisfactory prediction performance with the C-indexes of 0.849(95% CI: 0.781-0.917) and 0.931 (95% CI: 0.882-0.980) in two cohorts, respectively. The radiomics nomogram was more accurate in predicting OS in patients with nasal ENKTL than the other two models. Based on the radiomics nomogram, patients were categorized into low-risk and high-risk groups in two cohorts (*P* all < 0.05). The high-risk group defined by this nomogram exhibited a shorter OS.

**Conclusion:**

The Rad-score was significantly correlated with OS for nasal ENKTL patients. Moreover, the MRI-based radiomics nomogram could be used for risk stratification and might guide individual treatment decisions.

## 1 INTRODUCTION

Extranodal Natural killer/T-cell Lymphoma (ENKTL) is an uncommon subtype of highly malignant Non-Hodgkin Lymphoma (NHL) with unique clinical behaviors, such as primary involvement of the nasal cavity and nasopharynx, a high infection rate of Epstein-Barr Virus (EBV), and high incidence in East Asia and South America [1, 2]. ENKTL typically occurs in non-nodal sites, such as the nose, nasopharynx, oropharynx, Waldeyer’s ring, and parts of the upper aerodigestive tract [3]. More than two-thirds of ENKTL are localized at diagnosis, which is treated with radiation therapy with a 5-year survival rate of about 70% [4, 5]. Nowadays, the chemotherapy regimen based on L-asparaginase has significantly improved the survival outcome of advanced-stage or recurrent patients [6, 7], while the 5-year survival is only about 40% [8]. Data on the safety and efficacy of high-dose therapy and autologous hematopoietic cell transplantation are scarce [9]. Recent clinical trials have shown some clinical efficacy for the recurrent or refractory cases treated with immune checkpoint inhibitors, Histone Deacetylase (HDAC) inhibitors, anti-CD38 or anti-CD30 antibodies, and other drugs [10-13]. However, the ideal treatment approach for ENKTL has not been conclusively established. Therefore, accurate pretreatment prognostic prediction plays a crucial role in determining the survival outcomes and individualized treatment in ENKTL. Pre-treatment MRI examinations are frequently used to obtain morphological information, such as visual interpretation and assessment of tumor invasion, due to their superior soft-tissue resolution compared to CT scans. Therefore, MRI is recommended as a routine examination before treatment [14].

Currently, risk evaluation models for ENKTL primarily include the InternationalPrognostic Index (IPI) [15], Korean Prognostic Index (KPI) [16], Prognostic Index of Natural Killer Lymphoma (PINK) [17], and Nomogram-revised Risk Index (NRI) [18]. However, none of these models can accurately predict outcomes due to the inherent biological heterogeneity of the tumors, which limits their evaluative capability.

Radiomics, an emerging and promising field, offers a non-invasive approach to evaluate the tumor microenvironment and quantify spatial heterogeneity [19]. Features derived from medical imaging provide abundant information about the biological and physiological characteristics of tumors, facilitating the characterization of intratumoral heterogeneity [20]. Radiomic features can be extracted from standard imaging modalities, including ultrasonography, Computed Tomography (CT), Magnetic Resonance Imaging (MRI), and Positron Emission Tomography-Computed Tomography (PET/CT), which may reflect pathological, genetic, or clinical characteristics. Radiomics has been successfully applied in cancer detection, disease identification, and prognosis prediction across various cancer types, including lung, breast, nasopharyngeal, and genitourinary cancers [21–25]. Wang *et al.* discovered that radiomics features derived from PET images have the ability to predict the outcomes of ENKTL patients [26]. However, the potential of radiomics features extracted from MRI images to identify high-risk patients with nasal ENKTL has not been reported.

This study aims to explore the relationship between pretreatment MRI-based radiomics signatures and the prognosis of patients with nasal ENKTL. We aim to develop and validate a radiomics-based model to accurately identify high-risk patients for mortality.

To the best of our knowledge, this is the first study to investigate whether an MRI-based radiomics signature can identify patients with unfavorable outcomes in nasal ENKTL.

## 2 MATERIALS AND METHODS

### 2.1 Patient Selection and Follow-up

**Fig. (1):**
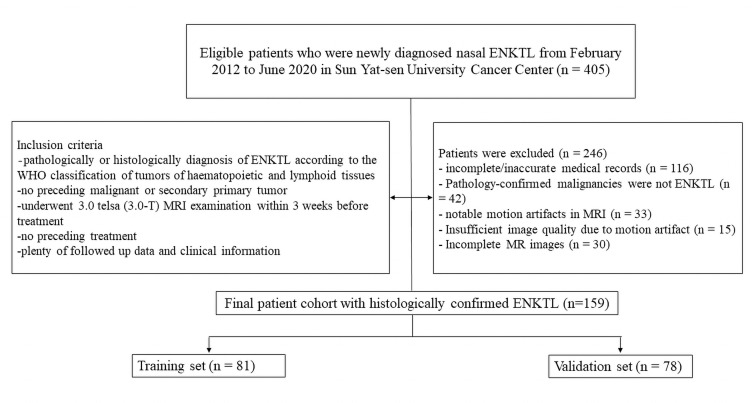
Flow diagram of the study enrolment patients.

The inclusion criteria were: (1) pathological or histological diagnosis of ENKTL; (2) no prior malignant or secondary primary tumor; (3) underwent 3.0 Tesla (3.0-T) MRI examination before treatment; (4) sufficient follow-up data and clinical information; and (5) evidence of measurable disease. The exclusion criteria were: (1) incomplete or inaccurate medical records and pretreatment MRI examinations; and (2) notable motion artifacts in MRI. Based on these criteria, 159 patients diagnosed with nasal ENKTL between February 2012 and June 2020 at Sun Yat-sen University Cancer Center were retrospectively included. These patients were randomly assigned into training (n=81) and validation (n=78) cohorts in approximately a 1:1 ratio (Fig. **1**).

The follow-up time was measured from the beginning of treatment to the last examination or death. After treatment, follow-up examinations were conducted generally every 3 months in the initial 3 years, every 6 months in the first 4-5 years, and annually thereafter until death. The end-point of this study was overall survival (OS), defined as the time from the date of diagnosis to the occurrence of death.

### 2.2 MRI Scan Acquisition

All patients underwent a pretreatment 3.0 T MRI scan (Signa EXCITE and Signa HDx, General Electric Healthcare, Chalfont St. Giles, United Kingdom; SIEMENS Espree, Siemens Healthcare, Erlangen, Germany) within 3 weeks before treatment. T1-weighted (T1-w) and T2-weighted (T2-w) images were acquired prior to the administration of contrast material, while contrast-enhanced T1-weighted (CET1-w) images were obtained after contrast injection. Imaging parameters are detailed in the supplementary materials.

### 2.3 Tumor Segmentation and Radiomics Feature Extraction

T2-w and CET1-w images in the Digital Imaging and Communications in Medicine (DICOM) format from the picture archiving and communication system (PACS) were utilized without any preprocessing or normalization. A radiologist with over 15 years of experience performed the tumor three-dimensional manual segmentation using 3D Slicer software (version 4.11.0, https://www.slicer.org). The Region of Interest (ROI), covering the entire tumor, was depicted across all slices of the coronal T2-w and CET1-w images on each slice. A radiologist with 20 years of experience then verified the accuracy of the segmentation.

The segmentation data were then imported into 3D Slicer to extract radiomics features. We used the 3D Slicer Extension Manager to install the Radiomics extension, which provides a graphical user interface for the PyRadiomics platform [27]. A total of 2,690 radiomics features were extracted from the ROI of each patient. The Intraclass Correlation Coefficient (ICC) was then used to assess the intra- and interobserver reproducibility of feature extraction. Features with an ICC greater than 0.7 were selected for further analysis, resulting in a final set of 2,074 features. Of these, 1,037 features were derived from T2-weighted (T2-w) images and 1,037 from contrast-enhanced T1-weighted (CET1-w) images. These features were categorized into four groups, as detailed in the supplementary materials.

### 2.4 Radiomics Feature Selection and Model Construction

The two-step feature selection method consisted of the two-sample t-test, and the logistic regression model with The Least Absolute Shrinkage and Selection Operator (LASSO) in the training cohort was employed to select features by Python software (version 3.9.0). LASSO Cox regression algorithm, a data analysis method, performs both variable selection and regularization of high-dimensional data [28]. The tuning regularization parameter λ, which determines the strength of regularization, was selected using tenfold cross-validation based on the minimum criteria. In this way, most covariate coefficients were reduced to zero, with the remaining non-zero coefficients employed by LASSO. A radiomics signature was then constructed using the selected features with non-zero coefficients [29]. The radiomics score (Rad-score) for each patient was calculated using a linear combination of the selected feature value, weighted by their corresponding non-zero coefficients.

### 2.5 Model Building and Validation

We calculated the Rad-score, defined as the radiomics signature, representing the combination of radiomics features selected in this study. Univariate and multivariate Cox regression analyses were performed to select significant clinical elements for OS prediction. The selected factors were put into a clinical model. We further developed a radiomics nomogram that integrated both Rad-score and clinical variables for OS prediction. To identify the optimal cutoff value for the radiomics nomogram-defined score, we utilized X-tile software (version 3.5.1). According to the cutoff values, patients were categorized into high-risk and low-risk groups. The potential predictive capability of the radiomics nomogram for OS was demonstrated in the training cohorts and subsequently validated through Kaplan-Meier survival analysis in different groups.

### 2.6 Statistical Analysis

The statistical analyses were performed by R software (version 4.0.2) *via* the packages and SPSS (version 23.0). Clinical characteristics between the two cohorts were compared using the Chi-square test. Univariate and multivariate Cox proportional hazard regression analyses were performed using SPSS to identify independent prognostic elements for OS. The hazard ratio (HR) and its 95% confidence Interval (CI) were calculated from the Cox regression analysis. Calibration curves, used to assess the accuracy of the nomogram, were plotted to compare the actual observed survival rates and the nomogram-predicted survival rates. Discrimination was measured by practicing Harrell's concordance index (C-index). The prognostic ability of the Rad-score, clinical model, and radiomics nomogram was further assessed using the Receiver Operating Characteristic (ROC) curves. The optimal radiomics nomogram-defined score value for predicting OS was determined by X-tile (version 3.6.1). A two-sided *P* < 0.05 was considered to be statistically significant. The process is presented in Fig. (**2**).

## 3 RESULTS

### 3.1 Clinical Characteristics

The clinical characteristics are listed in Table **1**. There were no significant differences in clinical characteristics between the two cohorts. The median follow-up was 40.0 months (interquartile range (IQR) 14.5–56.0) and 34.5 months (IQR 11.0 – 57.5) in the training and validation cohorts, respectively. During the last follow-up, 19/81 (23.5%) and 23/78 (29.5%) patients had died in the two cohorts, with no statistical significance observed (*P* = 0.389).

### 3.2 Radiomics Feature Extraction, Selection, and Radiomics Signature Building

**Fig. (3):**
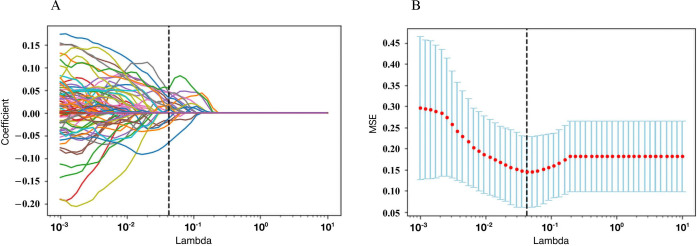
Radiomic feature selection using the least absolute shrinkage and selection operator (LASSO) regression model. Abbreviations: MSE, mean squared error.

After performing the intra-observer and inter-observer reliability analyses, 2,074 features with ICC scores > 0.7 were included: 1,037 features from T2-w images and 1,037 features from CET1-w images. Based on the two-sample t-test and LASSO analysis method (Fig. **3**), a final selection of 20 features was made for building the radiomics signature, with 11 features selected from T2-w images and 9 features selected from CET1-w images. These selected features had non-zero coefficients for OS. The optimal regulation weight (λ) of 0.0429 was employed under the minimum criterion. The 20 selected features included 2 features derived from the first-order metrics of the histogram and shape, 4 features derived from the Grey-Level Co-Occurrence Matrix (GCLM), 2 features derived from the grey-Level Size Zone Length Matrix (GLSZM), 1 feature derived from the neighborhood Gray-Tone Difference Matrix (NGTDM) and 11 features derived from the wavelet features (Table **S1**). These 20 features were selected for inclusion in the Rad-score, which was defined as the radiomics signature. The calculation formula of the Rad-score is shown in the Supplementary material. The Rad-score yielded a C-index of 0.733 (95% CI: 0.645 to 0.816) and 0.824 (95% CI: 0.766-0.882) in the two cohorts, respectively.

### 3.3 Model Building and Validation

**Fig. (4):**
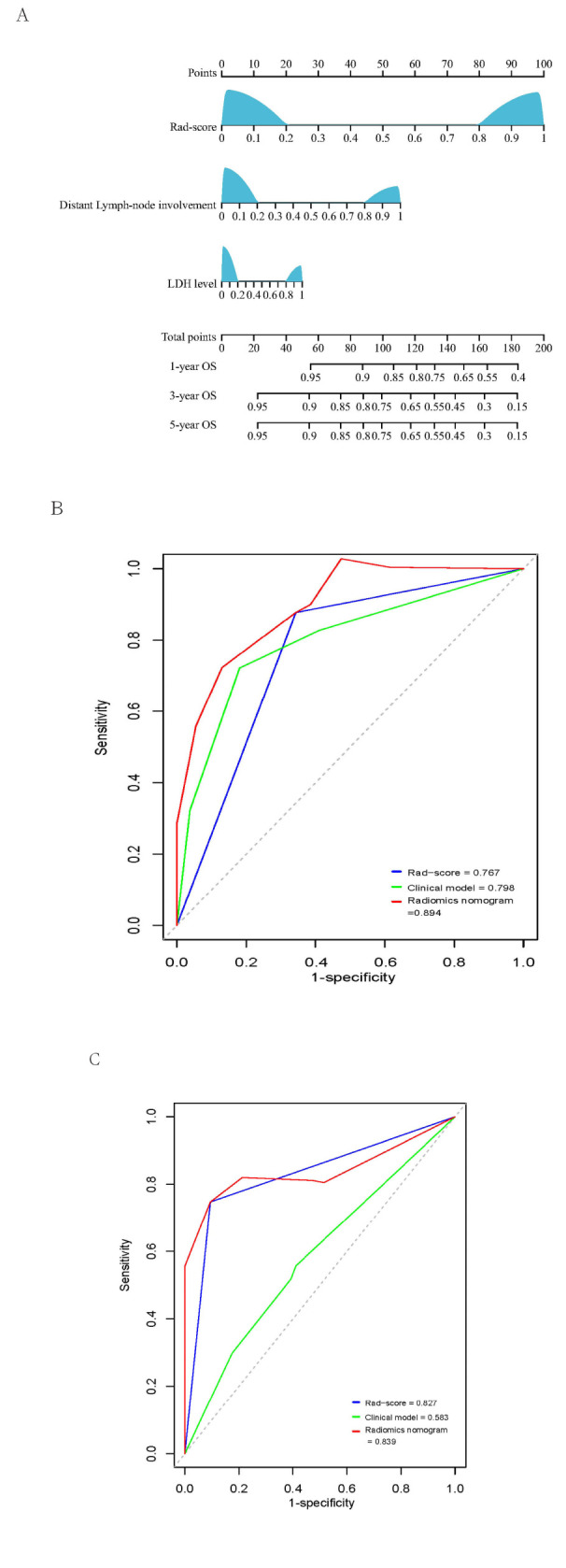
Nomogram based on the radiomics signature and clinical risk factors for predicting OS (**A**).; Receiver operating characteristic (ROC) curves comparing the predictive power of the radiomics nomogram, clinical model, and Rad-score for predicting OS in the training cohort, N = 81 (**B**); the validation cohort, N = 78 (**C**).

The results of the univariate and multivariate analyses for OS in the training cohort are listed in Table **2**. Through forward conditional Cox regression analysis, three independent risk factors were identified: Rad-score (HR: 10.962, 95% CI: 3.417-35.167, *P* < 0.001), lactate dehydrogenase (LDH) level (HR: 3.009, 95% CI: 1.128-8.510, *P* = 0.028), and distant lymph-node involvement (HR: 2.966,95% CI: 1.015-8.664, *P* = 0.047). According to the multivariate analysis results, patients with distal lymph node involvement and elevated LDH levels before treatment were included in the clinical model. The C-indexes of the clinical model for OS prediction were 0.707 (95% CI: 0.600–0.814) in the training cohort and 0.635 (95% CI: 0.527–0.743) in the validation cohort. The above factors, including Rad-score, LDH level, and distant lymph node involvement, were further combined to build a radiomics nomogram in the training cohort, as shown in Fig. (**4A**). The C-indexes of the radiomics nomogram for OS prediction were 0.849 (95% CI: 0.781-0.917) and 0.931 (95% CI: 0.882-0.980) in two cohorts, respectively. The prognosis ability of these models was compared using the ROC curve. The Area Under Curve (AUCs) of the three models were 0.767, 0.798, and 0.894 in the training cohort (*P* < 0.05, Fig. **4B**). When tested in the validation cohort, the AUCs were 0.827,0.583 and 0.839 (*P* < 0.05, Fig. **4C**). The calibration plot for the probability of OS showed excellent agreement between nomogram prediction and the actual observed OS (Fig. **S1**).

### 3.4 Survival Analysis Stratified by the Radiomics Nomogram

**Fig. (5):**
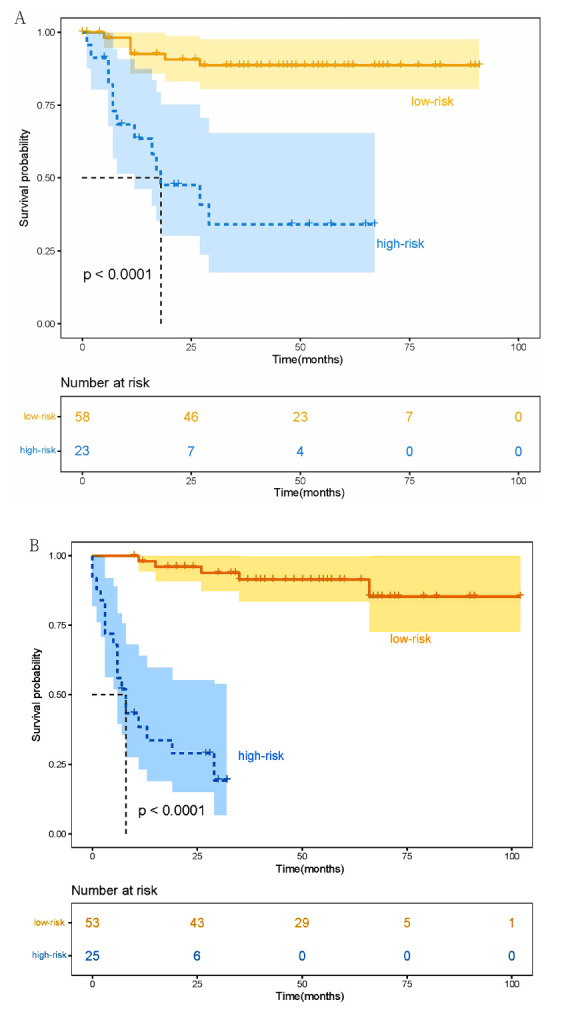
Kaplan-Meier curves of OS between high-risk and low-risk groups stratified by the radiomics nomogram for patients in the training cohort, N = 81 (**A**); the validation cohort, N = 78 (**B**).

The optimal cutoff value for the radiomics nomogram-defined score in predicting OS was determined to be 0.589 in the training cohort, and then the patients were categorized into high-risk and low-risk groups. In the training cohort, 23 patients (28.4%) were classified into the high-risk group, while 25 patients (32.1%) were assigned to the high-risk group in the validation cohort. Conversely, 58 patients (71.6%) and 53 patients (67.9%) were assigned to the low-risk group in the training and validation cohorts, respectively. In the training cohort, significant differences in OS were observed between the high-risk and low-risk patients, as shown in Fig. (**5A**) (1-year OS rates: 60.9% *vs*. 86.2%; 2-year OS rates: 30.4% *vs*. 79.3%, *P* < 0.001). These results were also validated in the validation cohort, where significant differences in OS were observed between the high-risk and low-risk groups (1-year OS rates: 32.0% *vs*. 94.3%; 2-year OS rates: 24.0% *vs*. 83.0%, Fig. **5B**, *P* < 0.001). Additionally, the IPI and KPI scores were evaluated in the current study to assess prognosis (Fig. **S2**). However, the IPI and KPI scores did not demonstrate the ability to effectively discriminate prognosis (*P* = 0.93 and 0.12, respectively). The representative images of the low-risk and high-risk cases are shown in Fig. (**S3**).

## 4 DISCUSSION

Radiomics is a promising new field in oncology that utilizes a high-throughput extraction method to transform medical images into high-dimensional, mineable data to obtain disease information to guide medical decisions in a non-invasive manner [30, 31]. Previous studies have demonstrated that MRI-based radiomics features have been applied for the diagnosis, prediction of treatment response, prognosis estimation, or other phenotypes [32-36]. Specifically, several studies have investigated MRI-based radiomics machine learning algorithms for identifying glioblastoma and primary central nervous system lymphoma [37], squamous cell carcinoma and lymphoma of the oropharynx [38], ocular adnexal lymphoma and idiopathic orbital inflammation [39] and so on. However, there is a lack of radiomics-based models for predicting the prognosis of Extranodal Natural Killer/T-Cell Lymphoma (ENKTL), with only Wang *et al.* proving that radiomics features derived from PET images can predict patient outcomes [26]. Nonetheless, PET examinations are expensive, and their images have relatively low spatial resolution and high noise, which may influence lesion identification [40].

In contrast, MRI has emerged as a vital tool for clinical diagnosis and therapeutic evaluation of nasal ENKTL due to its superior soft tissue contrast compared to CT scans. Therefore, in this study, we innovatively explored the MRI-based radiomics features performance in predicting OS of nasal ENKTL. To the best of our knowledge, this is the first study to incorporate prognostic features based on MRI radiomics to enhance the prognosis prediction for ENKTL, and significantly, this study was validated in an independent validation cohort (79 cases).

In this study, we introduced a multiparametric MRI-based radiomics signature, defined as the Rad-score, as a new approach to individually evaluate Overall Survival (OS) before treatment in nasal ENKTL. Furthermore, we developed and validated a radiomics nomogram that integrated the Rad-score and clinical variables, which exhibited superior performance compared with the radiomics signature and clinical model, and provides an easily accessible, non-invasive method for risk assessment in patients with ENKTL. To begin, we extracted comprehensive features from T2-weighted (T2-w) and contrast-enhanced T1-weighted (CET1-w) images. Only 20 potential descriptors were selected by using the two-sample t-test selection method and the LASSO logistic regression model. These 20 radiomics features can reflect different aspects of structural and potential tumor biological information. A total of 4 features were derived from the GCLM, which quantifies the probability that the adjacent gray scales with the highest frequency appear in pairs in ROI. A smaller probability indicates a more complex texture pattern. A total of 2 features were derived from the GLSZM, reflecting the volume uniformity in ROI. The larger the value, the better the regional uniformity. Further, 1 feature was derived from first-order metrics of the histogram and shape, which refer to gray-level statistical information in the ROI (including average value, entropy, and homogeneity) and describe the overall distribution of image intensity [19]. Another feature was derived from the NGTDM, which characterizes texture consistency and spotty or aperiodic texture. NGTDM demonstrated better performance than particle size, gray-level run-length matrix, and gray-level co-occurrence matrix for analyzing nuclei, dermis, road quality (asphalt road), and PET image texture [41]. Finally, 11 features were derived from the wavelet features. The undecimated three-dimensional (3D) wavelet transform was used to resolve the original image, which can be regarded as preprocessing prior to feature extraction [19].

The radiomics signature demonstrated satisfactory discrimination in the two cohorts (C-index = 0.733 and 0.824, respectively). One possible reason for this is the remarkable spatiotemporal heterogeneity of tumors at various levels: genes, proteins, cells, microenvironments, tissues, and organs. This heterogeneity offers promising prospects for tumor imaging evaluation [19, 42]. Radiomics features are typically extracted from intratumoral regions, reflecting biological characteristics related to tumor heterogeneity [43]. Imaging techniques can quantify spatial changes in tumor structure and function, measure phenotypic characteristics such as blood flow, hypoxia, metabolism, and cell death, and map the spatial distribution of biochemical pathways and cell signaling networks by quantifying basic biophysical parameters, such as MRI signal relaxation rates [44, 45].

According to the results of the univariate and multivariate analyses, we included age, Ann Arbor stage, and distant lymph node involvement in the clinical model, which have already been validated retrospectively in several previous multicenter studies [16-18]. The C-indexes of the clinical model were 0.707 and 0.635, respectively. Some recent studies attempted to construct a radiomics nomogram by combining the radiomics signature with the TNM stage or other parameters, which established a better progression-free survival (PFS) or OS prediction [24, 46]. Accordingly, to enhance the power of the decision support model and provide a clinically applicable method, we further combined the Rad-score with significant clinical risk factors to develop an effective radiomics nomogram, which achieved better predictive values (C-index = 0.849 and 0.931) than that of the Rad-score and clinical model. These findings offer new perceptions of future treatment strategies for patients. Future studies are needed to validate our findings.

With the development and application of new imaging devices, contrast agents, standardized scanning protocols, and multimodal imaging techniques, quantitative imaging of tumors can be performed [30]. Radiomics, a field combining medical imaging, genetic analysis, and clinical data through artificial intelligence methods, provides quantitative analysis of pixel uniformity in images and offers more information for clinical applications than traditional imaging methods. However, accurately classifying tumor areas and conducting radiomics analysis remain major challenges [47]. Further prospective, randomized studies are warranted to verify our conclusion.

The limitations of the present study are as follows. First, this is a single-center, retrospective study with a small sample size, which may have affected patient selection and radiomics quantification results. Future prospective studies with larger samples are necessary to confirm the conclusion of the current study. Secondly, the tumor three-dimensional segmentation was accomplished manually, which is time-consuming and complicated. An automatic segmentation method with higher reliability and reproducibility is needed. Finally, the stability of the radiomics nomogram needs further improvement by employing a larger training set with multicenter enrollment using different MRI protocols.

## CONCLUSION

In conclusion, we have identified and validated a 20-feature Rad-score as a powerful prognostic tool for nasal ENKTL patients. The radiomics nomogram, which integrates the Rad-score and clinical risk factors, provides a convenient approach for accurately predicting individualized OS. Our results may influence the treatment decision-making process.

## Figures and Tables

**Fig. (2):**
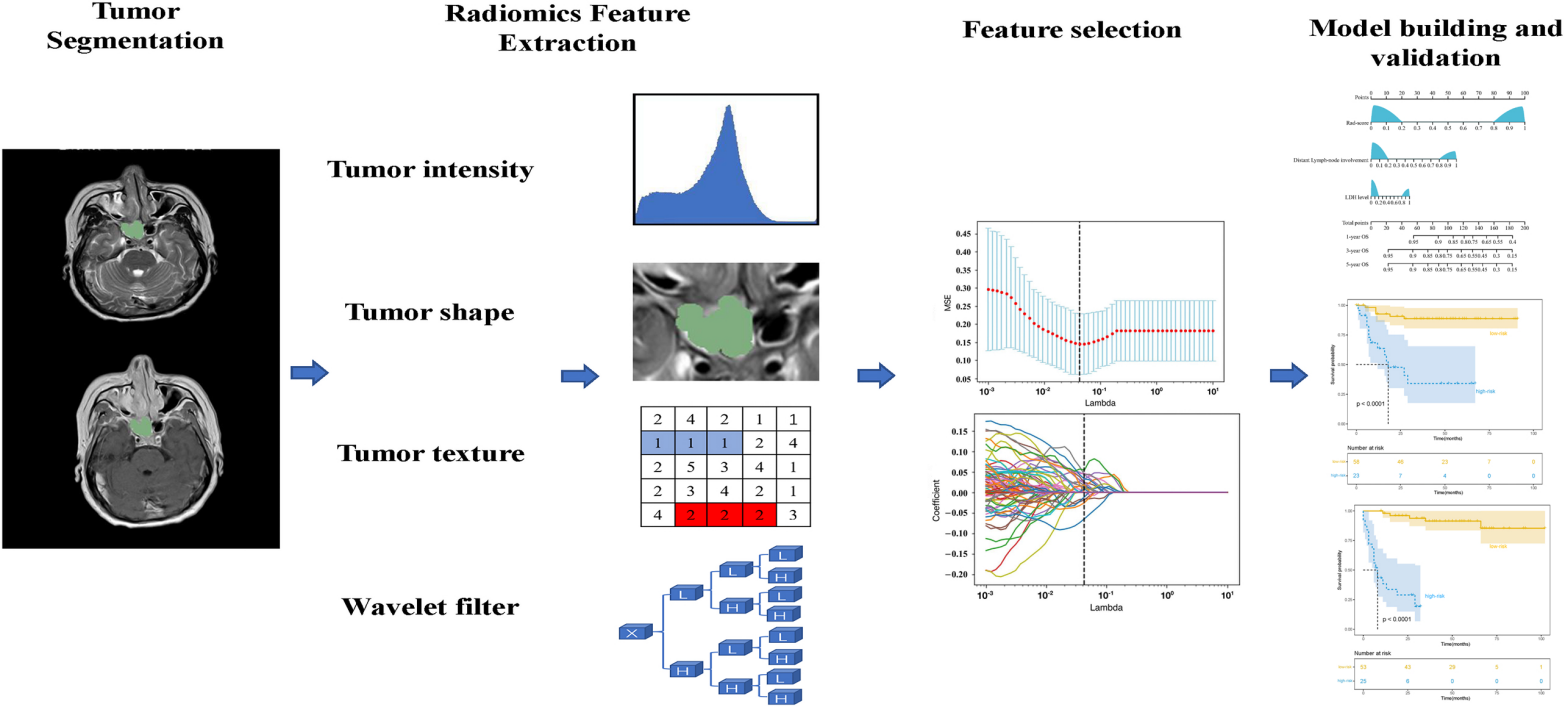
Flowchart showing the process of radiomics.

**Table 1:** Baseline characteristics of patients.

**Characteristics**	**Training Set**		**Validation Set**		**P-value**
	**No. of Patients**	**%**	**No. of Patients**	**%**	
**Age**	-	-	-	-	-
<60	74	91.4	68	87.2	0.394
≥60	7	8.6	10	12.8	-
**Gender**	-	-	-	-	-
Male	55	67.9	50	64.1	0.613
Female	26	32.1	28	35.9	-
**KPS score**	-	-	-	-	-
≤80	2	2.5	18	23.1	<0.01
>80	79	97.5	60	76.9	-
**Ann Arbor stage**	-	-	-	-	-
I–II	71	87.7	61	78.2	0.113
III–IV	10	12.3	17	21.8	-
**B symptoms**	-	-	-	-	-
Yes	38	46.9	49	62.8	0.044
No	43	53.1	29	37.2	-
**LDH level**	-	-	-	-	-
Elevated	25	30.9	19	24.4	0.359
Normal	56	69.1	59	75.6	-
**Hemophilic syndrome**	-	-	-	-	-
Yes	1	1.2	7	9	0.062
No	80	98.8	71	91	-
**Extranodal involvement sites**	-	-	-	-	-
<2	46	56.8	38	48.7	0.308
≥2	35	43.2	40	51.3	-
**Skin involvement**	-	-	-	-	-
Yes	5	6.2	7	9	0.504
No	76	93.8	71	91	-
**Distant lymph node involvement**	-	-	-	-	-
Yes	26	32.1	34	43.6	0.135
No	55	67.9	44	56.4	-
**EBV infection**	-	-	-	-	-
Yes	55	67.9	52	66.7	0.868
No	26	32.1	26	33.3	-
**Paranasalsinus invasion**	-	-	-	-	-
Yes	21	25.9	24	30.7	0.498
No	60	74.1	54	69.3	-
**IPI score**	-	-	-	-	-
0~1	62	76.6	56	71.8	0.494
2~5	19	23.4	22	28.2	-
**KPI score**	-	-	-	-	-
0~1	52	64.2	49	62.8	0.857
2~5	29	35.8	29	37.2	-
**Received chemotherapy**	-	-	-	-	-
Yes	80	98.8	77	98.7	1
No	1	1.2	1	1.3	-
**Received radiotherapy**	-	-	-	-	-
Yes	61	75.3	49	62.8	0.088
No	20	24.7	29	37.2	-

**Abbreviations**: KPS score, Karnofsky score; LDH, lactate dehydrogenase; EBV, Epstein-Barr virus; IPI score, International Prognostic Index score; KPI score, Korean Prognostic Index score.

**Table 2:** Univariate and multivariate analyses of potential prognostic factors for OS using the training cohort.

**Characteristics**	**Univariate Analysis**		**Multivariate Analysis**	
	**HR (95% CI)**	**P-value**	**HR (95% CI)**	**P-value**
**Age**				
<60	1	**0.021**	1	0.784
≥60	3.659(1.212-11.046)		1.187(0.348-4.051)	
**Gender**				
Male	1	0.903		
Female	1.062(0.403-2.796)			
**KPS score**				
<80	1	0.305		
≥80	0.348(0.046-2.613)			
**Ann Arbor stage**				
I–II	1	**0.008**	1	0.977
III–IV	4.022(1.434-11.280)		1.019(0.279-3.726)	
**B symptoms**				
No	1	0.868		
Yes	1.080(0.438-2.658)			
**LDH level**				
elevated	1	**0.001**	1	**0.028**
normal	0.211(0.085-0.527)		3.009(1.128-8.510)	
**Extranodal involvement sites**				
<2	1	0.078		
≥2	2.315(0.911-5.882)			
**Skin involvement**				
No	1			
Yes	1.803(0.416-7.808)	0.431		
**EBV infection**				
No	1	0.141		
Yes	2.523(0.735-8.664)			
**Paranasalsinus invasion**				
No	1	0.193		
Yes	1.859(0.730-4.729)			
**Distant lymph-node involvement**				
No	1	**0.001**	1	**0.047**
Yes	4.766(1.859-12.217)		2.966(1.015-8.664)	
**Rad-score**				
<147.14	1	**<0.001**	1	**<0.001**
≥147.14	12.314(4.611-32.884)		10.962(3.417-35.167)	

**Abbreviations**: KPS score, Karnofsky score; LDH, lactate dehydrogenase; EBV, Epstein-Barr virus.

## Data Availability

The data of current study are available from corresponding author, [C.G], on a reasonable request.

## LIST OF ABBREVIATIONS

OS: Overall Survival
NHL: Non-Hodgkin Lymphoma
KPI: Korean Prognostic Index
IPI: InternationalPrognostic Index
NRI: Nomogram-revised Risk Index
PINK: Prognostic Index of Natural Killer Lymphoma
MRI: Magnetic Resonance Imaging
CT: Computed Tomography
ROI: Region of Interest
C-index: Concordance index
IQR: Interquartile range
GCLM: Grey-Level Co-Occurrence Matrix
PFS: Progression-free survival

## STANDARDS OF REPORTING

STROBE guidelines were followed.
