# Supplementary material for: Development of a Radiomic-clinical Nomogram for Prediction of Survival in Patients with Nasal Extranodal Natural Killer/T-cell Lymphoma
Source: Curr Med Imaging. 2025 Jun 19;21:e15734056319914. doi: 10.2174/0115734056319914250605053257 (PMC13176757; doi:10.2174/0115734056319914250605053257)
Supplement: Supplementary file 1 [file CMIM-21-E15734056319914_SD1.pdf]

1573-4056/25 *Send Orders for Reprints to* reprints@benthamscience.net

BENTHAM SCIENCE
CrossMark

Current Medical Imaging

Content list available at: https://benthamscience.com/journals/cmir

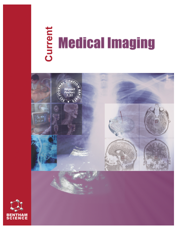

Development of a Radiomic-clinical Nomogram for Prediction of Survival in Patients with Nasal Extranodal Natural Killer/T-cell Lymphoma

Limin Chen[1,#], Zhao Wang[1,#], Xiaojie Fang[1,#], Mingjie Yu[1], Haimei Ye[1], Lujun Han[2], Ying Tian[1], Chengcheng Guo[3,*] and Huang He[1,*]

[1]*Department of Medical Oncology, Sun Yat-sen University Cancer Center, State Key Laboratory of Oncology in South China, Collaborative Innovation Center for Cancer Medicine, Guangdong Provincial Clinical Research Center for Cancer, Guangzhou 510060, Guangdong, China*
[2]*Department of Radiology, Sun Yat-sen University Cancer Center, State Key Laboratory of Oncology in South China, Collaborative Innovation Center for Cancer Medicine, Guangdong Provincial Clinical Research Center for Cancer, Guangzhou 510060, Guangdong, China*
[3]*Department of Neurosurgery, Sun Yat-sen University Cancer Center, State Key Laboratory of Oncology in South China, Collaborative Innovation Center for Cancer Medicine, Guangdong Provincial Clinical Research Center for Cancer, Guangzhou 510060, Guangdong, China*

Article History	Received: March 04, 2025	Revised: March 10, 2025	Accepted: April 10, 2025

Table S1. Selected radiomics features and coefficients.

Feature name	Coefficient
T2-w-GLCM-Idn	0.032
T2-w-GLCM-inverse variance	-0.016
T2-w-first-order-kurtosis	0.043
T2-w-first-order -contrast	-0.019
T2-w-GLCM-maximum probability	0.00008
T2-w-GLCM- zone variance	0.023
T2-w-wavelet-LLH-GLSZM-Gray Level nonuniformity	0.010
T2-w-wavelet-LLH-GLSZM- zone variance	0.046
T2-w-wavelet-HLH-GLCM- Idn	0.017
T2-w-wavelet-HLH-GLCM- maximum probability	0.032
T2-w-wavelet-LLL-GLCM- difference entropy	-0.011
CETI-w-NGTDM- busyness	0.022
CETI-w-GLSZM- large area low gray level emphasis	0.009
CETI-w-GLRLM- short run low gray level emphasis	-0.025
CETI-w-wavelet-LLH- GLSZM- zone variance	0.043
CETI-w-wavelet-LHL-GLDM- dependence nonuniformity	0.018
CETI-w-wavelet-HHL-NGTDM- busyness	0.011
CETI-w-wavelet-LLL-first-order- skewness	-0.064
CET1-w - wavelet-LLL-GLDM-Gray Level nonuniformity	0.054
CET1-w - wavelet-LLL-GLSZM- Gray Level nonuniformity	0.008

Abbrestions: T2-w, T2-weighted; CE-T1-w, contrast-enhanced T1-weighted; GLCM, the grey-level co-occurrence matrix; GLSZM, the grey-level size zone length matrix; NGTDM, the neighborhood gray-tone difference matrix; GLRLM, the gray-level run-length texture matrix; GLDM, the gray-level different matrix.

DOI: 10.2174/0115734056319914250605053257, 2025, *21*, e15734056319914

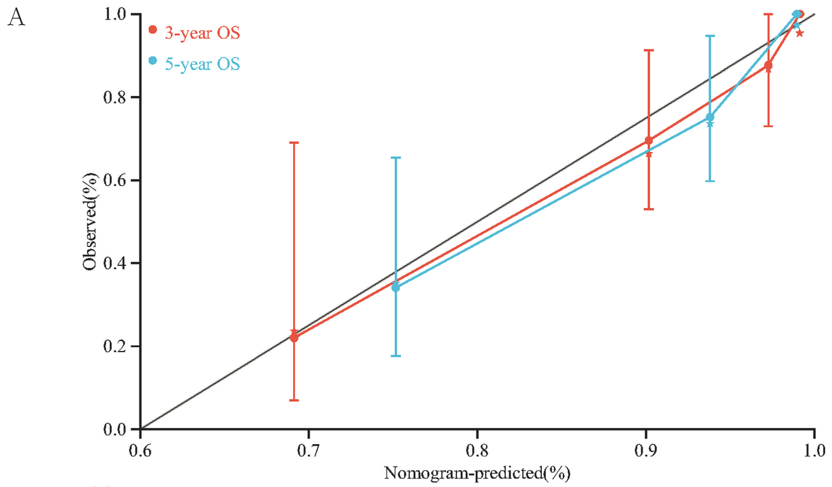

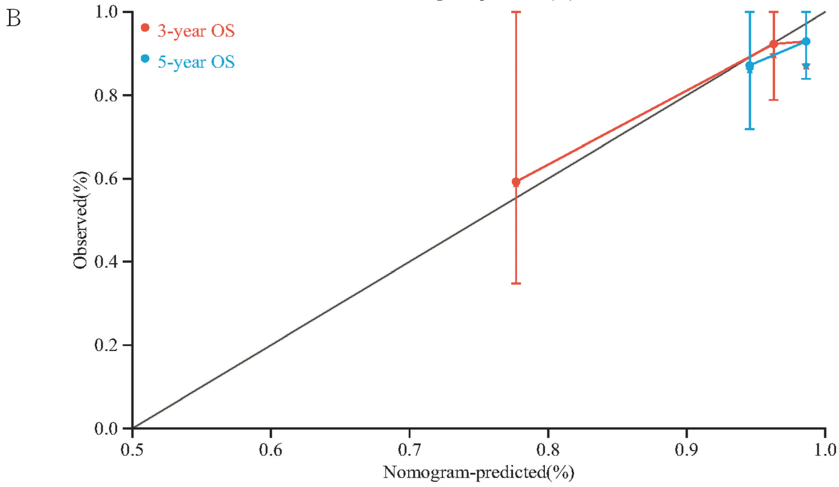

Fig. (S1). Receiver operating characteristic (ROC) curves comparing the predictive power of the radiomics nomogram, clinical model, and Rad-score for predicting OS in the training cohort, N = 81 (**A**); the validation cohort, N = 78 (**B**).

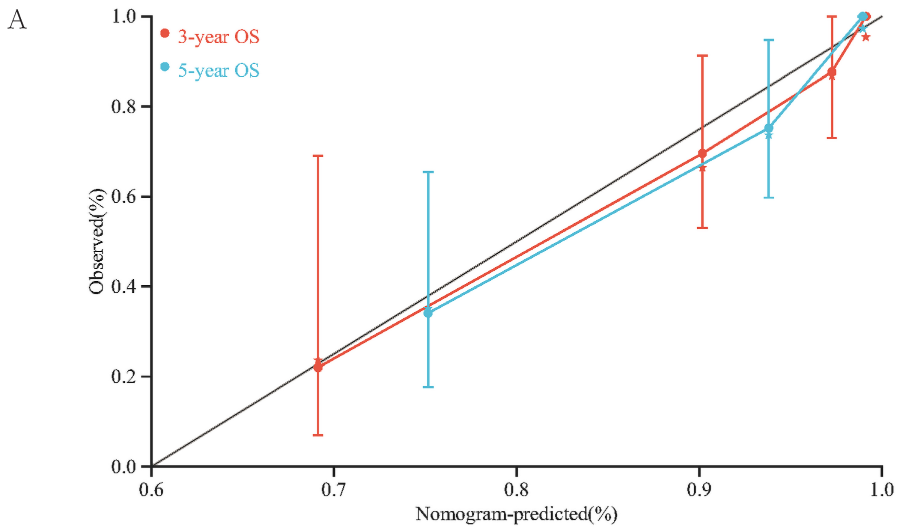

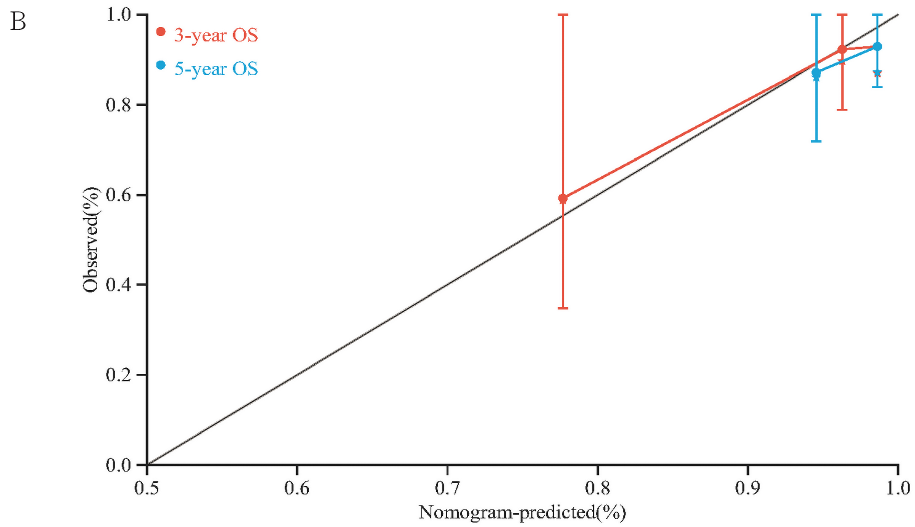

Fig. (S2). Risk stratification and overall survival according to IPI (**A**) and KPI (**B**) for patients of the training cohort.

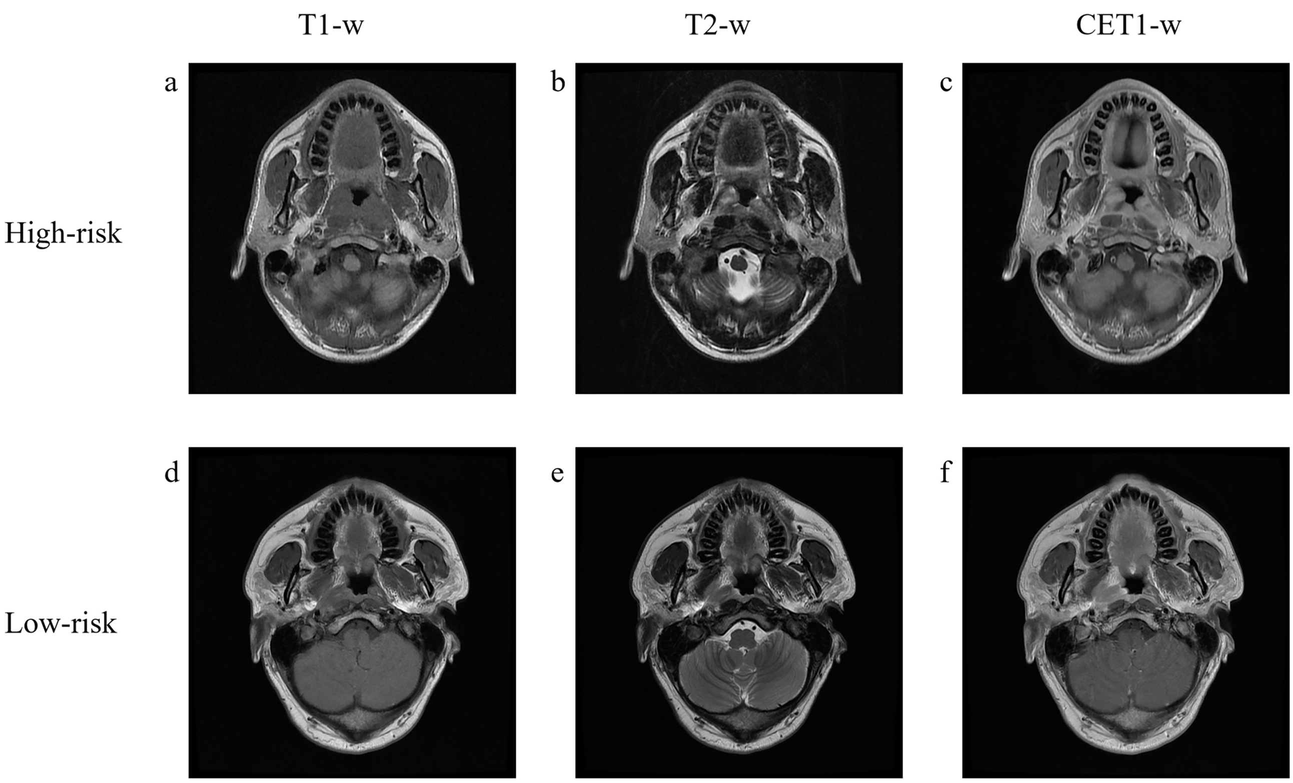

Fig. (S3). Representative MR images of the high risk (**a**,**b**,**c**) and low risk cases (**d**,**e**,**f**) in T1-weighted (T1-w), T2-weighted (T2-w) and contrast enhanced T1-weighted (CET1-w) sequences.

© 2025 The Author(s). Published by Bentham Science Publisher.

This is an open access article distributed under the terms of the Creative Commons Attribution 4.0 International Public License (CC-BY 4.0), a copy of which is available at: https://creativecommons.org/licenses/by/4.0/legalcode. This license permits unrestricted use, distribution, and reproduction in any medium, provided the original author and source are credited.
